# Smoking and degenerative spinal disease: A systematic review

**DOI:** 10.1016/j.bas.2022.100916

**Published:** 2022-08-07

**Authors:** Niharika Rajesh, Jigishaa Moudgil-Joshi, Chandrasekaran Kaliaperumal

**Affiliations:** a Royal College of Surgeons in Ireland, 123 St Stephen’s Green, Dublin, D02 YN77, Ireland; b College of Medicine and Veterinary Medicine, University of Edinburgh, 49 Little France Crescent, Edinburgh, UK; c Department of Neurosurgery, Oxford University Hospitals NHS Foundation Trust, Oxford, UK; d Department of Clinical Neurosciences (DCN), 50 Little France Crescent, Edinburgh, UK

## Abstract

Smoking is a major cause of morbidity and mortality worldwide and is responsible for the death of more than 8 million people per year globally. Through a systematic literature review, we aim to review the harmful effects of tobacco smoking on degenerative spinal diseases (DSD). DSD is a debilitating disease and there is a need to identify if smoking can be an attributable contender for the occurrence of this disease, as it can open up avenues for therapeutic options. Sources such as PubMed and Embase were used to review literature, maintaining tobacco smoking and spinal diseases as inclusion factors, excluding any article that did not explore this relationship. Risk of bias was assessed using analysis of results, sample size and methods and limitations. Upon review of the literature, tobacco smoking was found to be a major risk factor for the occurrence of DSDs, particularly lumbar spinal diseases. Smokers also experienced a greater need for surgery and greater postoperative wound healing complications, increased pain perception, delay in recovery and decreased satisfaction after receiving surgery. These effects were noted along the entire spine. Many mechanisms of action have been identified in the literature that provide plausible pictures of how smoking leads to spinal degeneration, exploring possible primary targets which can open up opportunities to develop potential therapeutic agents. More studies on cervical and thoracic spinal degeneration would be beneficial in identifying the effect of nicotine on these spinal levels. Some limitations included insufficient sample size, inconclusive evidence and lack of sufficient repeat studies. However, there appears to be a sufficient amount of research on smoking directly contributing to lumbar spinal pathology.

## Highlights

• Smoking is a risk factor for the occurence of degenerative spinal disease (DSD).
• There are numerous pathological mechanisms attributed to spinal pathology by smoking.
• Smoking appears to be a significant risk factor for lumbar DSDs, with smoke studies also suggesting its role in cervical DSDs.
• There is insufficient research on the effect of smoking on the thoracic spine.
• Smoking leads to worse outcomes and potential complications post-surgery, as well as increased pain perception and poorer subjective response post-surgery.

## Abbreviations/acronyms:

DDD: Degenerative spinal disease
IVD: Intervertebral disc
DDD: Degenerative disc disease
NP: Nucleus Pulposus
AF: Annulus Fibrosis
ADAMTS: Activation of a disintegrin and metalloproteinase with thrombospondin motifs
IGD: Interglobular domain
TSE: Tobacco smoke extract
VNTR: Variable number of tandem repeat
CEP: Cartilage end-plates
TUD: Tobacco use disorder
LDH: Lumbar Disc Herniation

## 1 Introduction

Smoking has been implicated as a risk factor or causative factor in a plethora of diseases afflicting most organ systems in the body. It is a major cause of morbidity and mortality worldwide and is responsible for the death of more than 8 million people per year globally (Tobacco, 2021). As a result of such widespread afflictions, smoking adds a major economic burden to the healthcare industry. For instance, despite pioneering in smoking control, England faces about 78,000 deaths per year due to smoking, with the cost to the NHS being around £2.5 billion of the £14.7 billion per year of the economy (Tobacco commissioning support, 2022). The chemicals present in cigarettes, along with tobacco, can affect various systems and organs in the body.

Degenerative diseases of the spine, particularly those of intervertebral discs (IVD) and those pertaining to the cervical and lumbar spine, are chronic diseases that are relatively common in occurrence. Several risk factors are associated with degenerative spine diseases (DSDs), amongst which smoking has been found to be an increasingly common factor. Studies have shown that cigarette smoking can contribute to the occurrence of certain DSDs, and has also been shown to not have a significant impact in others.

There is a strong contention for smoking being a risk factor for spinal stenosis as showcased through multiple studies (Bagley et al., 2019; Abbas et al., 2013; Ding et al., 2021; Sheung-tung, 2017; Sandén et al., 2011; Stienen et al., 2016; Gulati et al., 2015). The effect of smoking on spinal stenosis can also be seen as the progression of spinal stenosis to spondylolysis, which is a fracture of the pars interarticularis and further progression into spondylolisthesis, which is the anterior displacement of the vertebrae that were affected (Gagnet et al., 2018). Furthermore, Degenerative disc disease (DDD) or IVD degeneration has also been found to be affected by cigarette smoke, regardless of where in the spine the degeneration may be occurring, although it appears that there may be a greater chance of the lumbar discs being affected by smoking (Bagley et al., 2019; Abbas et al., 2013; Ding et al., 2021; Sheung-tung, 2017; Sandén et al., 2011; Stienen et al., 2016; Gulati et al., 2015).

**Fig. 1:**
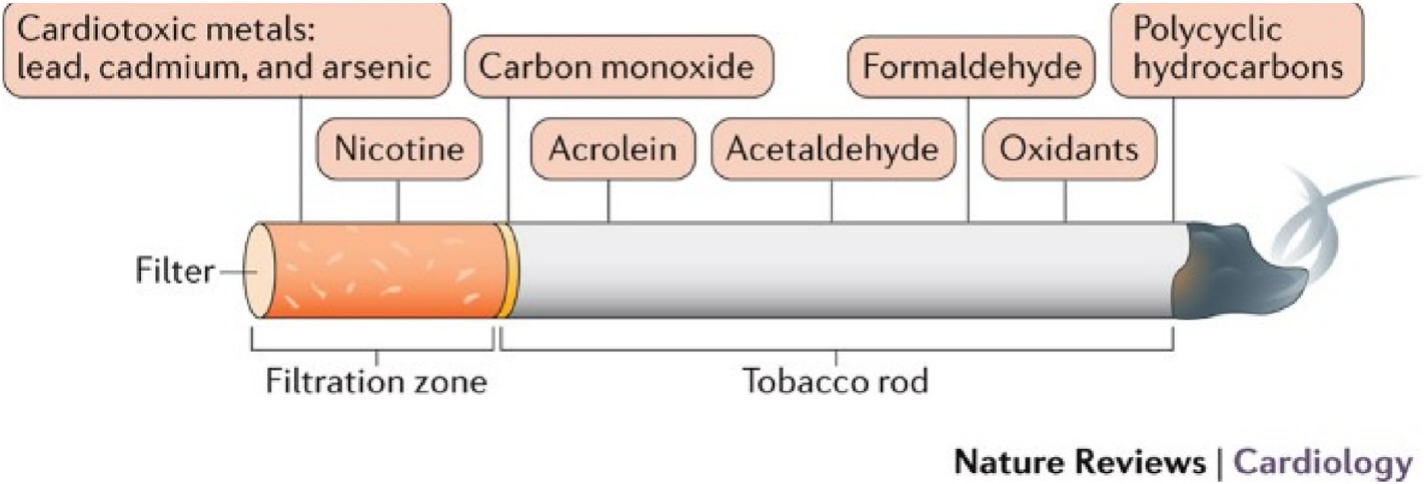
Image showing the composition of a cigarette, with the chemicals present being extremely toxic and harmful to the human body (Benowitz and Fraiman, 2017).

Cigarette smoke contains nicotine, which has been found to be a main player when it comes to DSD. There have been several theories about the mechanisms by which chemicals in tobacco smoke induce spinal injury and degeneration, including the upregulation or downregulation of certain genes that may be critical to maintain the integrity of the spine. Apart from nicotine, some other toxic chemicals present in cigarettes include cardiotoxic metals i.e. cadmium, lead, nickel and chromium, carbon monoxide, formaldehyde, acrolein, acetaldehyde, oxidants that can stimulate reactive oxygen species formation and polycyclic hydrocarbons, which cause endothelial damage and subsequent formation of atherosclerotic plaques (Fig. 1) (Benowitz and Fraiman, 2017).^,^ (Caruso et al., 2013)

In this review we discuss the relevant literature and pathophysiological aspects focussing on the effects of tobacco smoking on DSD.

## 2 Methods

### 2.1 Search strategy

Various databases and platforms were used to conduct a literature search for this review. Google Scholar, PubMed, Embase and Web of Science were used to conduct advanced searches. The following keywords were used to conduct a thorough search of the literature:

**Table:** 

	Search terms used
1.	“Degenerative spinal disease” AND (“smoking” OR “tobacco”)
2.	“Cervical spinal stenosis” AND (“smoking” OR “tobacco”)
3.	“Lumbar spinal stenosis” AND (“smoking” OR “tobacco”)
4.	“Thoracic spinal stenosis” AND (“smoking” OR “tobacco”)
5.	“Degenerative disc disease” AND (“smoking” OR “tobacco”)
6.	(“Degenerative disc disease” OR “cervical disc disease” OR “lumbar disc disease” OR “thoracic disc disease” OR “disc prolapse”) AND (“smoking” OR “tobacco”))
7.	(“Spondylosis” OR “Spondylolisthesis”) AND (“smoking” OR “tobacco”)
8.	“Cervical myelopathy” AND (“smoking” OR “tobacco”)

Duplicate articles were disregarded from the search, along with those that did not explore DSDs in the context of smoking. Any possible articles that examined this correlation were used in this paper and were grouped according to the subheadings listed. Table .1 summarises the key words used for literature search.

**Table 1:** Table compiling literature used for the review.

Key:	
Lumbar Spinal Stenosis	LSS
Degenerative Spine Disease	DSD
Degenerative Disc Disease	DDD
Intervertebral disc	IVD
Thoracic Spinal Stenosis	TSS
Nucleus Pulposus	NP
Annulus Fibrosus	AF
Collagen End Plate	CEP
Lumbar Disc Herniation	LDH

## 3 Results

### 3.1 Pathological effects of smoking on a molecular level

There are a few explanations on how cigarette smoke and its constituents cause damage on a molecular level. One such explanation is that nicotine, a component of cigarettes, works directly on osteoblastic cells, preventing cellular proliferation, metabolism and collagen synthesis, whilst also reducing bone density and blood supply to the bone (Sharma and Petrukhina, 2013). Hence, these smokers have a greater susceptibility to bony degradation and can consequently develop DSD as a result (Sharma and Petrukhina, 2013). High levels of primary tobacco inhalation promote degeneration of the vertebral bone and discs, even if only exposed for a short period of time (Nasto et al., 2014). Nicotine has additionally been found to induce vasoconstriction, which can affect the spinal cord, causing decreased perfusion, poor nutrition and potential medullary ischaemia (Baucher et al., 2021; Elmasry et al., 2015). This can cause the barrier between the vascular supply and spinal cord to disintegrate, leading to oedema as a result of increased permeability (Baucher et al., 2021; Elmasry et al., 2015). Elmasry et al. reported similar findings of reduced nutrients and anabolic agents being provided to the IVD, and also further reported damage to glycosaminoglycans biosynthesis in the IVD, which contributes to its instability and degeneration (Elmasry et al., 2015). According to Fogelholm and Alho, cigarette smoke induces the release of elastase and other proteases into circulation from neutrophils in pulmonary capillaries. It is further worsened by the fact that cigarette smoke inhibits the protease inhibitor, alpha-1-antiprotease. Thereby, proteolytic activity in the spine is still present when unrequired, causing excessive damage (Nasto et al., 2014).

Smoking was also found to primarily exert its effects on degenerative cervical myelopathy by impacting IVD degeneration (Baucher et al., 2021). The pathophysiology could potentially be due to cigarette smoke activating genes that upregulate proinflammatory stress responses and induce dose-dependent cell toxicity in the nucleus pulposus (NP) and annulus fibrosus (AF) (Baucher et al., 2021). This can cause cell death and degeneration due to a metabolic imbalance brought on by the stress response (Baucher et al., 2021).

**Fig. 2:**
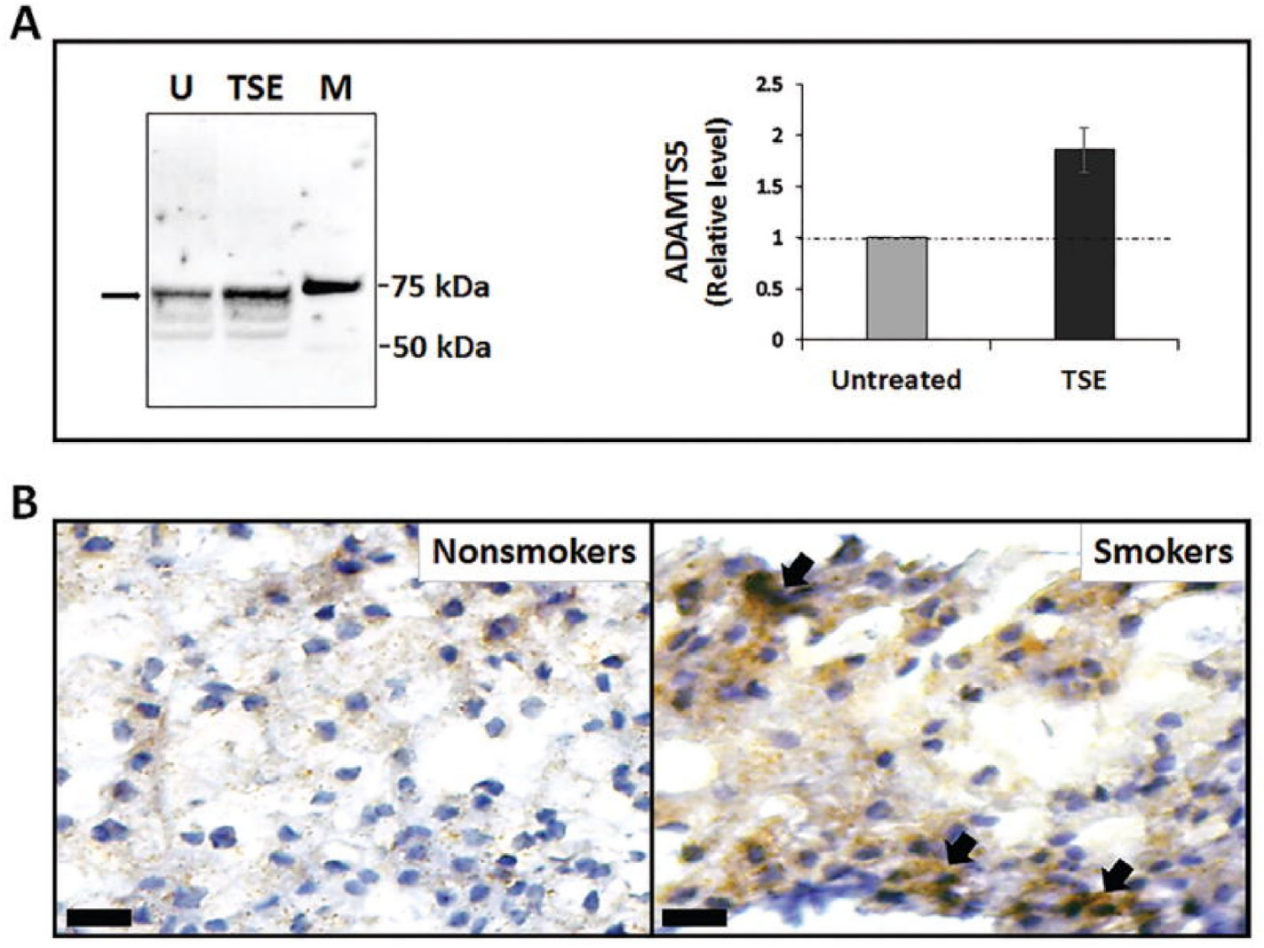
(A) Left: Western blot showing ADAMTS5 protein levels in untreated (U) versus TSE treated samples with an obvious increase in TSE samples; M represents protein markers (Ngo et al., 2017). **Right:** Quantitation of the 73 ​kDa band from the Western blot by densitometry. **(B)** Immunohistochemical detection of ADAMTS5 in the NP of mice that were unexposed (non-smokers) and exposed (smokers) to tobacco smoke (Ngo et al., 2017).

**Fig. 3:**
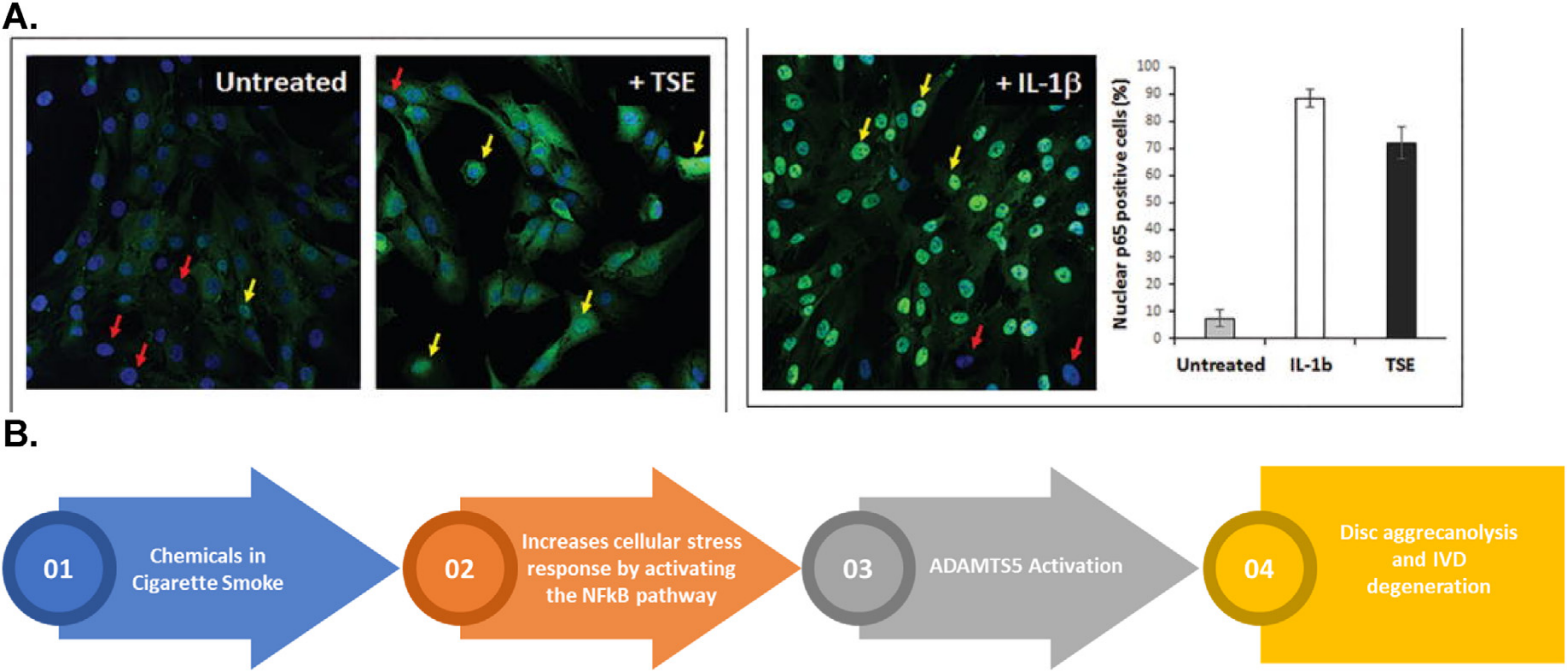
(A) Immunofluorescence showing the levels of nuclear p65, a subunit of NF-κB in untreated vs TSE treated samples, thus showing increased NF-κB activity (Ngo et al., 2017). **Red arrows:** Absent nuclear p65; **Yellow arrows:** Presence of nuclear p65; IL-1β is the positive control. Right image is a quantification of the Immunofluorescence data. **(B)** Stepwise pathway showcasing mechanism of action of IVD degeneration due to smoking (Ngo et al., 2017). (For interpretation of the references to colour in this figure legend, the reader is referred to the Web version of this article.)

Several articles have explored the possibility of genetic changes brought upon by cigarette smoke contributing to DSD development. Smoking could induce degeneration of the spine through cellular DNA damage, at least to a certain extent, as was found by observing the effects on mice with certain knockout genes (Nasto et al., 2014). A mechanism of action for IVD degeneration was explored by Ngo et al. looking at proteoglycan loss, a hallmark of IVD degeneration, due to tobacco smoke induced DNA changes (Nasto et al., 2014; Ngo et al., 2017). This occurs alongside damage caused by the free radicals, inflammatory compounds, and genotoxins induced by cigarette smoke (Sharma and Petrukhina, 2013). Using mice models, they showed that tobacco smoking causes the activation of a disintegrin and metalloproteinase with thrombospondin motifs (ADAMTS) gene, particularly ADAMTS5. This protein cleaves the interglobular domain (IGD) of disc aggrecan, a major proteoglycan in the articular cartilage that allows for a hydrated gel structure, which causes the release of the aggrecan (Ngo et al., 2017; KIANI et al., 2002). This is considered pathological as it compromises the integrity of the IVD. On Western blot, the ADAMTS5 protein levels were found to be increased in samples treated with tobacco smoke extract (TSE); this was subsequently quantified by densitometry (Fig. 2A left and right images respectively) (Ngo et al., 2017). Immunohistochemical detection of ADAMTS5 was also shown (Fig. 2B) (Ngo et al., 2017). This was further compared with NF-κB activity as it appeared to be the confounder that was increased by smoking, which in turn induced ADAMTS5 activation (Fig. 3A) (Ngo et al., 2017). A schematic summarizing the potential mechanism of action has been included (Fig. 3B) (Ngo et al., 2017).

Another association was examined between the aggrecan variable number of tandem repeat (VNTR) polymorphism and disc degeneration amongst the Chinese Han in northern China (Cong et al., 2010). This association had been previously studied in the Finnish population (Cong et al., 2010). This study examined the relationship between smoking and aggrecan gene VNTR in causing IVD degeneration by studying the MRI images of patients. In terms of repeats, the study found that participants with ≤25 repeats who did not smoke showed a 1.102-fold increased risk for symptomatic IVD degeneration (p ​= ​0.855, 95% CI 0.389–3.119), those with two alleles >25 repeats who smoked for more than 1 pack year had a 1.013-fold increase in risk (p ​= ​0.982, 95% CI 0.333–3.084) and those with one or two alleles ≤25 repeats who smoked more than 1 pack-year had a 4.5 fold increase in risk of IVD degeneration (p ​= ​0.005, 95% CI 1.589–12.743) (Cong et al., 2010). This showed a potential pathway by which smoking could lead to disc degeneration (Cong et al., 2010).

**Fig. 4:**
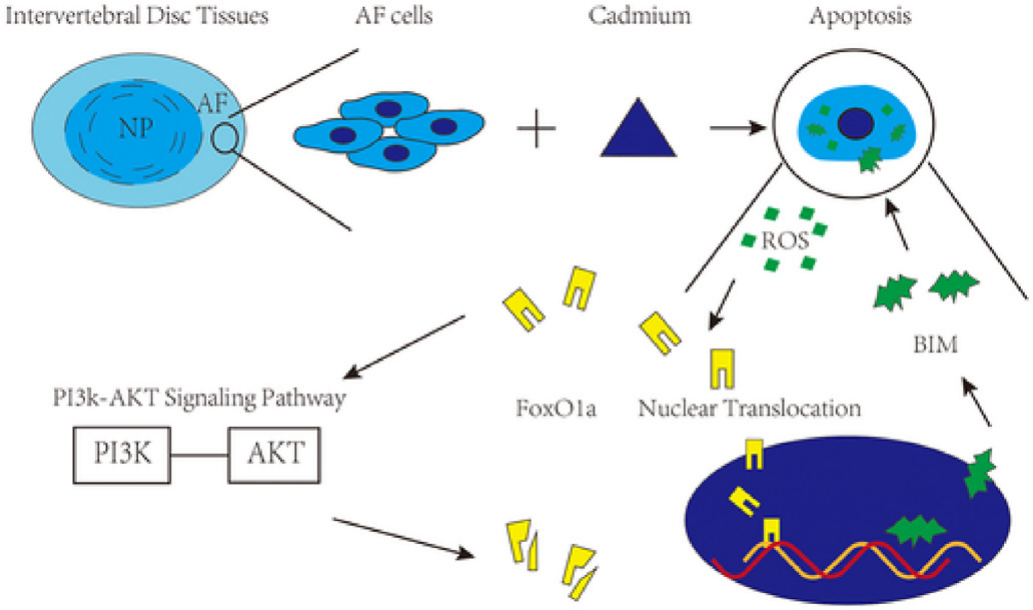
Schematic showing the mechanism of action of cigarette smoke on AF cell apoptosis, and subsequent IVD degeneration through the activation of FOXO1a, as well as the counter mechanism of action by the PI3K/AKT pathway (Jing et al., 2020).

Jing et al. reported another gene that could potentially be targeted to prevent the negative effects of smoking on the spine. Smoking caused accumulation of cadmium in the body, which induced the apoptosis of AF cells and in turn caused IVD degeneration (Jing et al., 2020). This event was caused by the nuclear translocation of the gene FOXO1a by cadmium, which activates the mitochondrial-related pathway to induce apoptosis of AF cells. Additionally, it was found that this process also involved the Phosphatidylinositol 3-kinase/protein kinase B (PI3K/AKT) signal pathway. The PI3K/AKT pathway led to the phosphorylation of FOXO1a, which reduced the percentage of AF apoptosis (Jing et al., 2020). This occurs in conjunction with the actions of FOXO1 activation (Jing et al., 2020). However, with increased exposure to cigarette smoke, there is more damage than protection to the IVD (Jing et al., 2020). A schematic of the possible mechanism of action can be found below (Fig. 4) (Jing et al., 2020).

**Fig. 5:**
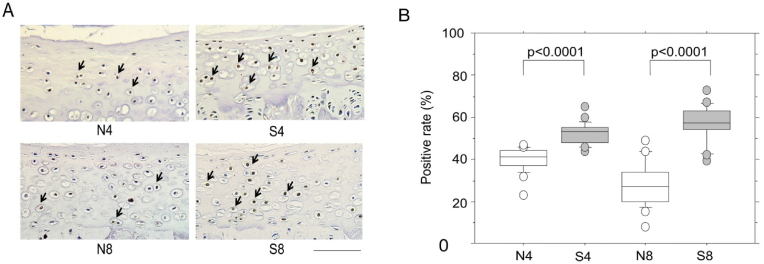
Apoptosis of CEP induced by passive smoking. (A) Immunostaining non-smoking (N) and smoking (S) rats for 4 or 8 weeks (eg. N4 or N8) for ssDNA of CEP. Arrows indicate cells containing ssRNA positive brown nuclei (Nakahashi et al., 2019). **(B)** The positive rate was calculated using the ssDNA positive cells in the CEP. A significant increase can be seen in smokers in both 4 and 8 weeks compared to non-smokers (Nakahashi et al., 2019). (For interpretation of the references to colour in this figure legend, the reader is referred to the Web version of this article.)

**Fig. 6:**
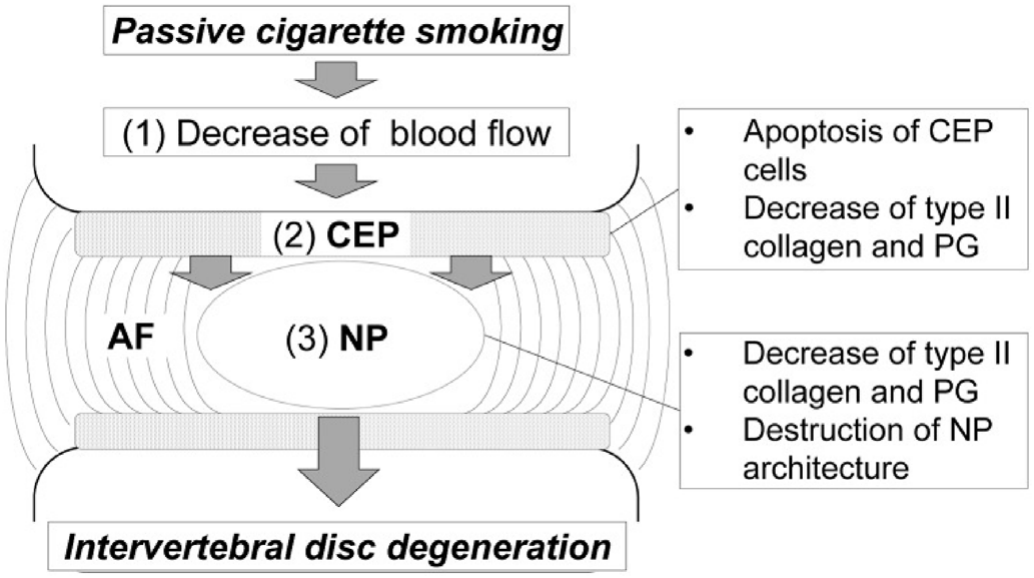
Schematic of a potential mechanism of action by which smoking leads to IVD degeneration (Nakahashi et al., 2019).

Nakahashi et al., through experiments using mice models, deduced a potential vascular-based mechanism of action by which smoking induced IVD degeneration (Nakahashi et al., 2019). Passive smoking was found to significantly decrease blood flow to the IVD, causing major histological and structural changes in the NP (Nakahashi et al., 2019). This was further followed by increased apoptosis and destruction of type II collagen and proteoglycans, which are components of the cartilage end-plates (CEP) of IVDs in smokers compared to non-smokers (p ​= ​0.003) (Fig. 5) (Nakahashi et al., 2019). Significant damage to the NP was also found in smokers (p ​< ​0.0001), although the effect on AF was not found to be significant. A schematic of the proposed pathway can be found below (Fig. 6) (Nakahashi et al., 2019).

Numaguchi et al. elucidated the detailed molecular changes caused by passive smoking in IVDs (Numaguchi et al., 2015). Many genes were altered in the AF, CEP and NP, of which 7 genes were found to be related to the circadian rhythm (Numaguchi et al., 2015). In AF and CEP, the phase of 4 genes shifted forward 4–6 ​h, whereas two genes, *Nr1d1* and *Npas2*, related to NP shifted forward 3–6 ​h and Arntl and Dbp, also related to NP, had their circadian rhythm abolished (Numaguchi et al., 2015). This was found to cause a phase shift, which causes a feeling similar to jet lag (Numaguchi et al., 2015). This phase shift could cause the cells of the IVDs to also be affected, thereby unable to maintain proper levels of production and degradation, ultimately leading to IVD degeneration as a result of passive smoking (Numaguchi et al., 2015).

**Fig. 7:**
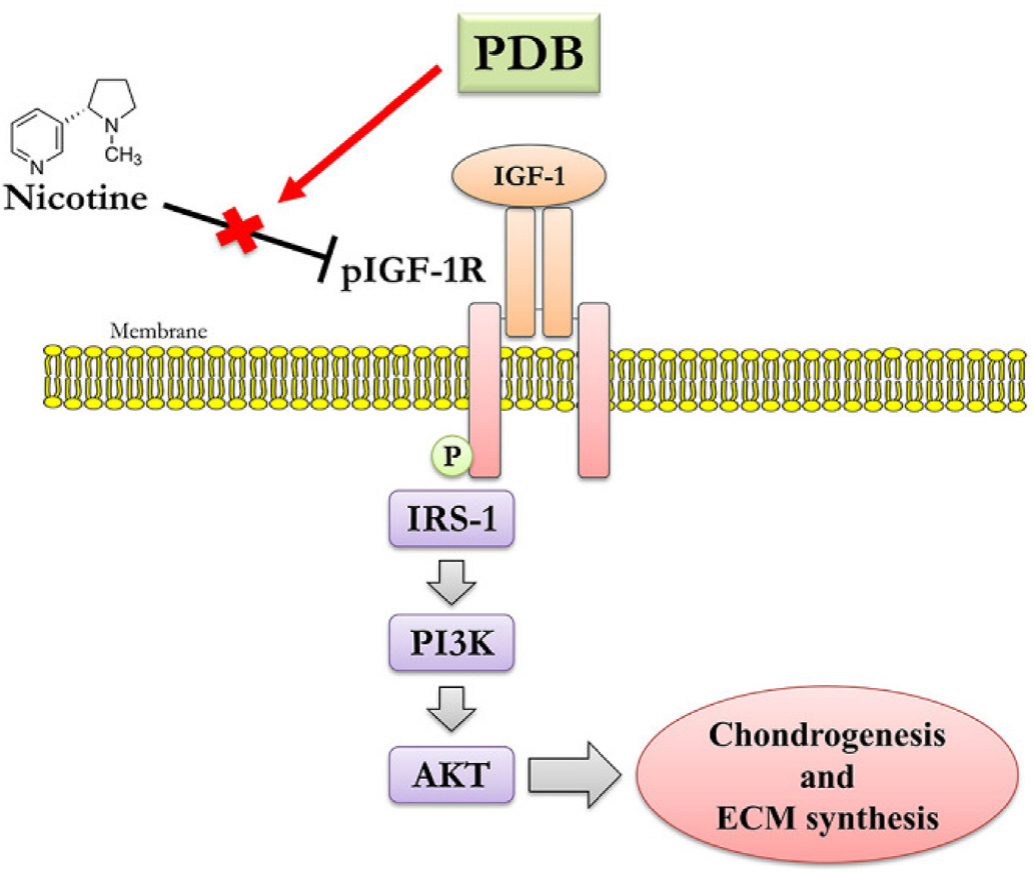
Schematic showing how nicotine leads to IVD degeneration via the improper synthesis of chondrocytes (Elmasry et al., 2015).

The article by Lo et al. explores the effect of nicotine on IVD degeneration by looking at how the chondrogenic indicators are affected. The indicators included Sox, Col II and aggrecan, all of which were found to be reduced, including the chondrocytes themselves following exposure to nicotine (P ​< ​0.001) (Lo et al., 2021). Furthermore, proteoglycan synthesis in healthy chondrocytes was found to be regulated by the IGF1/AKT regulatory machinery (Elmasry et al., 2015; Lo et al., 2021). Hence, it was hypothesised, and found, using a therapeutic target molecule (PDB), that nicotine significantly declined the phosphorylated levels of IGF-1, AKT and IRS-1, hence preventing proper proteoglycan synthesis, chondrocyte synthesis and maintenance of the IVD, leading to degradation (Elmasry et al., 2015). A schematic of this pathway is shown below (Fig. 7) (Elmasry et al., 2015).

**Fig. 8:**
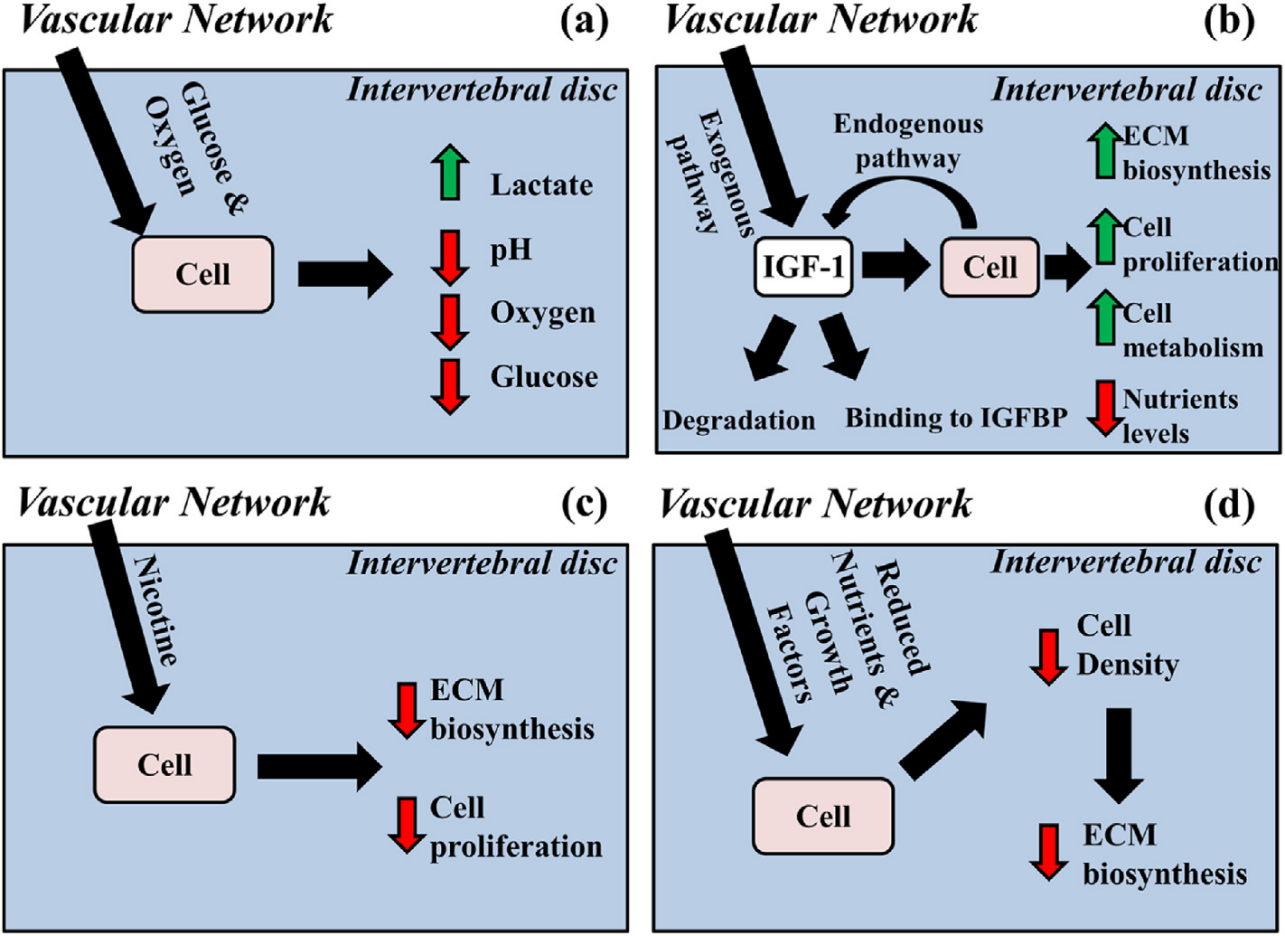
Schematic showing the vascular homeostasis of the IVD and how it is affected by changing nutrient levels and nicotine (Elmasry et al., 2015).

Interesting data on passive smoking causing vasoconstriction was also present, which reduces blood flow to the spine and thus leads to IVD degeneration (Holm and Nachemson, 1988; Bellitti et al., 2021). Overall, there appeared to be a great focus on mechanism of action of cigarette smoking on IVD, with studies on chondrocyte development as well as proteoglycan and glycosaminoglycan synthesis in the IVD, leading to poor development and maintenance of the discs (Elmasry et al., 2015). Furthermore, there appeared to be a common pattern of NP degeneration or AF and CEP degeneration in discs, with vascular supply being affected in many cases due to the vasoconstriction caused by nicotine (Fig. 8) (Elmasry et al., 2015).

Table .2 summarises the possible pathological mechanisms attributed to spinal pathology by smoking.

**Table 2:** Table summarizing pathologies leading to spinal degeneration.

Degenerative spinal disease	Possible pathological mechanisms
**Spinal Stenosis**	Nicotine has an adverse effect on osteoblastic cells, as a result of which smokers are more susceptible to bony degradation, resulting in degenerative disease of the spine (Hadley and Reddy, 1997).
	Poor wound healing and outcome following surgery for LSS, with increased risk of complications and infection was found. (Sheung-tung, 2017)
	Poor pain management and decreased outcomes were also observed for people with LSS exposed to cigarette smoke (Sandén et al., 2011).
	Smoking increased the risk of repeating surgery in patients who underwent surgery for LSS (Stienen et al., 2016).
	Smoking caused increased instability of the spine and increased risk of stenosis (Kwon et al., 2020).
**Degenerative cervical myelopathy**	Smoking upregulates proinflammatory genes, causing dose-dependent toxicity (Baucher et al., 2021).
	Vasoconstriction brought on by nicotine can also be responsible for the occurrence of degenerative spinal disease (Baucher et al., 2021).
	Smoking increased postoperative risk of complications following surgery for cervical myelopathy (Sheung-tung, 2017).
	Smoking decreased the range of motion in patients following surgery for cervical myelopathy, along with increasing the risk for reoperation (Liu et al., 2021).
	The risk of re-operation increased when tobacco use disorder was present in conjunction with cervical myelopathy (Liu et al., 2021).
**Spondylosis/Spondylolisthesis**	Smoking caused impairment of vascular and bone integrity, which caused spinal damage, leading to spondylosis, which can progress to spondylolisthesis (Khurana, 2021).
	Smoking status was a great predictor of risk of reoperation after lumbar laminectomy for lumbar spondylosis (Bydon et al., 2015).
	The spinal instability caused by smoking increased the risk of occurrence of spondylolisthesis (Kwon et al., 2020).
	Smoking caused wound complications and increased pain following certain surgeries for spondylosis and spondylolisthesis (Nakhla et al., 2018).
**IVD degeneration- Overall**	Upregulation of proinflammatory stress responses and the corresponding dose-dependent toxicity also causes degeneration of the IVD (Baucher et al., 2021).
	The vascular supply to IVD is critical, and the vasoconstriction caused by nicotine can lead to ischaemia and degeneration of the spinal discs. It also impairs oxygen tension due to reduced supply, and increases lactate levels (Holm and Nachemson, 1988).
	Nicotine was also found to affect GAG levels in the IVD, which is critical for the maintenance of the architecture of the disc. Hence, it causes degeneration of the IVDs (Elmasry et al., 2015).
	Proteoglycan levels in the disc, which are also important to maintain the integrity of the discs, were reduced due to nicotine. Smoking before and during pregnancy and lactation also caused increased fibrosis and decrease in proteoglycan amount, causing instability of the disc and increased degeneration (Altun and Yuksel, 2017).
	Passive smoking affects both NP and CEP of IVDs, where the components of NP are damaged whereas CEP undergoes apoptosis (Nakahashi et al., 2019).
	Smoking also causes a shift in the circadian rhythm of the body by acting on certain genes responsible for their control, which interferes with the proper functioning of certain molecular mechanisms, leading to IVD degeneration (Numaguchi et al., 2015).
	IVD degeneration due to smoking is increased by activating ADAMTS5, which causes pathological release of aggrecan and also induced inflammatory pathways, causing further disc damage (Ngo et al., 2017).
	Another method of degeneration is the accumulation of cadmium induced by smoking, which leads to increased apoptosis of AF, by activating the mitochondrial pathway and causing excessive release of ROS, with the involvement of the FoxO1a gene (Jing et al., 2020).
	Nicotine also causes degeneration by inhibiting IGF-1 release, which causes chondrocyte destruction (Lo et al., 2021).
**Lumbar DDD**	Smoking affected predominantly the L5-S1 spinal levels, causing increased degeneration, although it also affects L3/L4 (Kiraz and Demir, 2020).
	Increased neutrophil to lymphocyte ratio was found in people with Lumbar DDD who were smokers (Doğan et al., 2019).
	Smoking increased pain and decreased patient satisfaction in patients undergoing surgery for lumbar DDD (Smith et al., 2014).
**Cervical DDD**	Active smoking damages C1/2 and C6/7 primarily, causing increased neck and shoulder pain in patients. It accelerates cervical spine degeneration (Chen et al., 2018).
	Complications such as developing adjacent segment disease following total disc replacement for cervical DDD (Nunley et al., 2013).
	Smoking caused a poorer early fusion effect and affected the bones, leading to bone loss, which caused increased degeneration of the cervical spinal discs (Wang et al., 2021).
**LDH**	Smoking increases the risk of lumbar herniation and narrowing by increasing the instability of the spine by causing bone degeneration (Schumann et al., 2010).
	Individuals with certain polymorphisms within certain genes were more susceptible to the damage induced by cigarette smoke, leading to LDH. This was found in two studies: Luo et al. (Luo et al., 2020) and Yang et al. (Yang et al., 2019)

Abbreviations: LSS-; IVD-; DDD- ROS-; AF-; LDH-;NP-;CEP-; GAG-; ADAMTS5-.

## 4 Discussion

We discuss various clinical studies published so far and have divided them into smoking contributing to risk factors for spinal degenetrative disease and how it affects wound healing and recovery.

### 4.1 Tobacco smoking as a risk factor for spinal degenerative disease occurrence

Smoking was found to be a risk factor in the development of various DSDs (Kwon et al., 2020). Its involvement was prominent in spinal stenosis, with some evidence pointing to the development of lumbar spinal stenosis, degenerative disease and atherosclerosis, which can further contribute to the narrowing of blood vessels (Bagley et al., 2019; Sharma and Petrukhina, 2013; Bellitti et al., 2021). Additionally, apart from being a leading risk factor for lumbar spine disease, smoking is also associated with its early onset (Sharma and Petrukhina, 2013). Smoking was also found to permanently affect baseline values in patients with spinal disease. Current smokers, according to Snyder, B.M et al., with spinal stenosis were found to have significantly worse baseline functional health status than both former and those that never smoked (Kwon et al., 2020; Snyder et al., 2022).

Contrastingly, Abbas et al. showcase the lack of a significant relationship between smoking and lumbar spinal stenosis, with the rates of smoking in the groups presenting with and without stenosis being similar (P ​= ​0.574) (Abbas et al., 2013). Similarly, Ding et al. deny any relationship between thoracic spinal stenosis and smoking (Ding et al., 2021). For cervical spinal stenosis, smoking was associated more with post treatment complications as opposed to being a risk factor, although this was not the case for cervical or lumbar DDD (Baucher et al., 2021).

Smokers were at a significantly higher risk of developing DDD (Elmasry et al., 2015; Altun and Yuksel, 2017; Wang et al., 2012; Jakoi et al., 2017; Simmons et al., 1996; Saberi et al., 2009). An interesting study by Altun et al. showcased that maternal smoking before and during pregnancy decreased the ratio of proteoglycans in the IVD, whilst also increasing fibrosis, leading to degeneration of the spinal discs across the entire spine (Altun and Yuksel, 2017; Wang et al., 2012).

There are also several studies looking at the effect of smoking particularly on the cervical spinal discs. Cigarette smoking was found to be associated with higher incidence of and greater acceleration of the process of cervical DDD (Chen et al., 2018; Lambrechts et al., 2021). Chen et al. found that cigarette smoke affected the lower cervical discs (C4–C5 and C5–C6) to a greater extent than the upper cervical discs (C1–C2 and C3–C4) (P ​< ​0.05) (Chen et al., 2018), although Lambrechts et al. found the entirety of the cervical spine to be affected equally (Lambrechts et al., 2021).

An interesting study by Grisdela et al. describes that cervical DDD is found to sometimes occur in conjunction with tobacco use disorder (TUD) in patients (3.5%, 11,337 patients), in which patients are addicted to tobacco use (Grisdela et al., 2017). These patients would undergo surgery (6.9%) more often than patients who did not have TUD (3%). The complications and number of affected individuals were increased in those patients whose cervical DSD progressed to myelopathy (Grisdela et al., 2017). However, it has been difficult to establish a cause-effect relationship between TUD and cervical DDD (Grisdela et al., 2017). Contrastingly, Gore et al. found no significant difference in the development of cervical DDD between smokers and non-smokers (p ​= ​0.9314). MacDowell et al. also suggested that smoking does not affect the pain caused by existing cervical DDD (MacDowall et al., 2017). Smoking was also found to have no correlation with cervical spondylotic myelopathy according to some studies (Baucher et al., 2021; Zhong et al., 2021).

Several studies observed a greater number of smokers developing lumbar DDD compared to non-smokers (p ​< ​0.05) (Jakoi et al., 2017; Lambrechts et al., 2021; Kiraz and Demir, 2020; Doğan et al., 2019). This effect was further enhanced if multiple additional comorbidities co-existed, such as diabetes and obesity or if there was a strong family history present (Jakoi et al., 2017; Simmons et al., 1996). On the other hand, a twin study established that despite being genetically identical, there was an 18% increase in lumbar DDD in twins that smoked compared to their non-smoking counterparts throughout the entire lumbar spinal region (BATTIÉ et al., 1991).

Other studies also observed potential genetic susceptibilities, which when exposed to cigarette smoke can lead to the development of lumbar DDDs. A study published by Yang et al. explored the relationship between the gene cluster CHRNA5/CHRNA3, which encode the alpha5 and alpha3 subunits of the nicotinic acetylcholine receptors and were found to have a strong association with smoking (p ​< ​0.001), and the risk of lumbar disc herniation (LDH) (Yang et al., 2019). Upon analysis, it was found that certain genotypes within these clusters were even more likely to experience lumbar disc herniation compared to others (Yang et al., 2019). In the CHRNA3 cluster, the rs8040868 CT genotype had a 0.46-fold higher LDH than the rs8040868 TT genotype amongst men (OR ​= ​0.46, 95% CI 0.25–0.84, p ​= ​0.012) (Yang et al., 2019). Amongst the CHRNA5 haplotypes, “TACACCCG” and “TACACCCG” were found to greatly increase the risk of LDH (Yang et al., 2019). Thus, genetic factors seemed to also play a role in enhancing the effects of smoking on IVD disease. Similarly, Luo et al. found that there was a strong association between the rs591058 ​C/T polymorphism of the matrix metalloproteinase (MMP)-3 gene and an increased risk of developing lumbar disc herniation in a southern Chinese population (Luo et al., 2020).

One specific study found that smoking particularly affected the lumbar discs of the L5-S1 vertebrae in patients who smoked for more and less than 10 years (P ​= ​0.022, P ​= ​0.048) (Kiraz and Demir, 2020). However, in patients that smoked for more than 10 years, smoking was found to increase haemoglobin value in smokers (P ​= ​0.018) compared to non-smokers (P ​= ​0.009), suggesting increased haemoglobin production in chronic smokers, particularly those affected in the L3-L4 lumbar spinal discs (Kiraz and Demir, 2020). The authors, based on their study, defined this relationship as being explained by decreased oxygenation of the lumbar discs due to the increase in carboxyhaemoglobin formation as a result of smoking, which led to increased haemoglobin value (Kiraz and Demir, 2020). Smoking was also found to promote lumbar disc herniation, with the relative risk of association being found to be 1.27 (Huang et al., 2015).

In contrast, Schumann et al. reported that there was no clear dose-response relationship between smoking and lumbar disc disease, although they also reported that the odds ratio for lumbar disc herniation was significantly increased in medium and high smoking patients (Schumann et al., 2010). It was found that in smokers with intervertebral disc degeneration, no observable modic changes were found in the lumbar endplates compared to non-smokers (Saberi et al., 2009; Han et al., 2017).

Smoking was not found to be correlated with degenerative lumbar spondylolisthesis (Huang et al., 2015; Jacobsen et al., 2007).

### 4.2 Tobacco smoking affecting postoperative wound healing and recovery

Multiple studies have found a correlation between smoking and poor improvement, recovery or increased complications following spinal surgery for DSD. Smoking was found to complicate spinal stenosis surgery for patients and lead to worse outcomes post-surgery (Sheung-tung, 2017; Snyder et al., 2022). However, it was not found to be associated with reoperation for patients (Sandén et al., 2011). A study found that the patient-reported outcome following micro decompression for lumbar spinal stenosis, examined using a change in the Oswestry Disability Index (ODI) was significant in smokers (ODI: 17.3 points, 95% CI: 15.93–18.67, p ​< ​0.001) at 1 year (Gulati et al., 2015). There was no difference in the overall complication rate (p ​= ​0.34) or length of hospital stays for single level (p ​= ​0.99) or two-level (p ​= ​0.175) micro decompression between smokers and non-smokers. Spinal fusion surgeries, however, have been found to be less successful in smokers compared to non-smokers (Hadley and Reddy, 1997).

Smokers were further found by Sanden et al. to experience greater postoperative pain compared to non-smokers, gathered from reports by patients during their 2-year follow-up (p ​< ​0.001) (Sandén et al., 2011; Fogelholm and Alho, 2001). He also reports greater dissatisfaction amongst smokers following surgery for DSD (OR 1.79, 95% CI:1.51–2.12), more frequent use of analgesics (OR: 1.86, 95% CI: 1.55–2.23), and a lower quality of life (p ​< ​0.001). Additionally, Connor et al. report an increased rate of readmissions 90 days post-elective cervical and thoracolumbar spine surgery following DSD (odds ratio: 1.05, 95% CI: 1.03–1.07, p ​< ​0.0001). Other studies also found that tobacco use delayed wound healing, and caused increased postoperative pain, decreased satisfaction following surgery, and decreased quality of life, findings similar to Sanden et al. (Liu et al., 2021; Connor et al., 2020). Additionally, in patients undergoing cervical laminoplasty, there was a higher trend of revision surgery noted in smokers compared to non-smokers (An et al., 1994). The effect of cigarette smoke appears to be based on the procedure that is being conducted, but contrastingly to the above information, individuals who underwent non-instrumented lumbar spine surgery appeared to have a favourable response in terms of back pain and health-related quality of life in both smokers and non-smokers for up to 4.5 years post-surgery (Stienen et al., 2016). Similarly, Joswig et al. also found contradicting results that patient-reported outcome measures for pain, functional impairment, and health-related quality of life for lumbar degenerative disk disease were similar amongst smokers and non-smokers (Joswig et al., 2017). Smoking was also noted to not play a role in repeat procedures for symptomatic adjacent segment disease (Tu et al., 2019).

In terms of other DSDs, the risk of developing lumbar IVD prolapse (P ​= ​0.000011) and cervical disc disease (P ​= ​0.00064) was found to be significantly increased in current smokers compared to non-smokers in patients who had undergone surgery for cervical or lumbar radiculopathy (Burkhardt et al., 2020). The relative risk was found to be 3.0 for lumbar disc diseases and 3.9 for cervical disc diseases (Burkhardt et al., 2020). The association was also found to be viable when comparing current smokers to ex-smokers (P ​= ​0.00029 for lumbar disc disease and P ​= ​0.0025 for cervical disc disease) (Burkhardt et al., 2020). Smoking also increased pain in patients with spinal disease before, during and through the course of their treatment and recovery (p ​< ​0.001) (Asher et al., 2017). Smoking cessation, however, was found to improve pain in those afflicted with painful spinal diseases (Asher et al., 2017). Smoking was found to have an effect on the outcomes following surgery for spondylolisthesis (Patel et al., 2020; Goyal et al., 2020). Chan et al. assessed the impact of smoking on patients who were undergoing single segment surgery for grade 1 lumbar spondylolisthesis (Goyal et al., 2020). The ODI was assessed and was found to be similar between smokers and non-smokers (Goyal et al., 2020). Both groups were also found to exhibit similar improvement from baseline ODI (p ​< ​0.00), however, smokers were less likely to achieve a minimum clinically important difference in ODI compared to non-smokers (OR ​= ​1.45, 95% CI [0.16–12.95], p ​= ​0.74) (Goyal et al., 2020). Echt et al. reports that patients who underwent posterolateral fusion alone for spondylolisthesis did not have a greater risk of wound complications, although those who underwent interbody fusion noted a much greater risk of wound complications compared to non-smokers (Patel et al., 2020).

A few studies also investigated the effect of smoking on cervical or lumbar total disc arthroplasty for disc herniation or spondylosis (Khurana, 2021; Konovalov et al., 2021; Smith et al., 2014). Interestingly, one study (Konovalov et al., 2021) found that smokers had a better outcome following surgery in that they had a more preserved range of motion and less heterotopic ossification compared to non-smokers following level 1- and 2 cervical disc arthroplasty, after a 3.5 year follow-up (Konovalov et al., 2021). This showcased the possibility of a good surgical option for smokers (Konovalov et al., 2021). Another study, however, reported no significant difference in clinical outcomes between smokers and non-smokers following lumbar disc arthroplasty (Smith et al., 2014). The incidence of adverse events and survival probability was found to be similar as well (Smith et al., 2014). However, the study found that smokers had a greater likelihood of developing heterotopic ossification after lumbar disc arthroplasty (Smith et al., 2014).

The impact of smoking on outcomes following other spinal surgeries were also examined. Smith et al. (Nunley et al., 2013) found that patients who underwent lumbar discogram for back pain were more likely to follow up post-surgery if they were smokers (P ​= ​0.013). Additionally, smoking was found to be the strongest predictor of people coming in for reoperation for lumbar laminectomy, regardless of if it was for single-level or multilevel laminectomy, and for reoperation for progressive spinal degeneration (Nakhla et al., 2018).

Several studies also reported no significant difference in smokers versus non-smokers when it came to post-surgical outcomes. For instance, Asher et al. reported that there was no difference in efficacy of interventions for lumbar spinal disorders between smokers and non-smokers (Bydon et al., 2015). Smoking was also not found to be a predictive factor for complications following cervical myelopathy (Stienen et al., 2016)and was not found to contribute to the incidence of symptomatic adjacent disc disease following cervical total disc replacement (Tetreault et al., 2016). Wang et al. explored the effects of smoking on outcomes following a hybrid surgery consisting of an anterior cervical discectomy and fusion, with cervical disc replacement (Wang et al., 2021). They found that the current smoking group faced a poorer early fusion effect (P ​< ​0.001) and a 1-year fusion rate (P ​< ​0.035) (Smith et al., 2014). They also found that smoking exacerbated bone loss, but did not have a significantly different clinical outcome compared to former smokers and non-smokers (Behrend et al., 2012). Additionally, Goyal et al. also reported that there was no difference in outcome following lumbar decompression surgery between current smokers, former smokers and never smokers.

There has been a relatively clear effect established between smoking and post-surgical complications in patients with lumbar degenerative disease, as can be seen in the results above. The strength of this relationship lies in the fact that several studies show similar significant results in terms of increased postoperative pain, poorer health-related quality of life and patient dissatisfaction post-surgery. Additionally, with relatively large sample size and a strong inclusion and exclusion criteria, Gulati et al. added greater strength to the study. Table .3 summarises various studies with their conclusions.

**Table 3:** A summary of the studies included in this review and their associated conclusions.

Author(s)	Year of Publication	Type of spinal degeneration	Type of Study/Characteristics	Conclusion
Bagley et al.	2019	LSS	Comprehensive review of Lumbar spinal stenosis	Smoking is detrimental to recovery following surgery. (Bagley et al., 2019)
Sharma, M.K. and Petrukhina, E	2013	Lumbar DSD	Case-Control Study	Smoking is a strong risk factor for lumbar DSD, especially those with early onset lumbar DSD. (Sharma and Petrukhina, 2013)
Gore et al.	2006	Cervical DSD	Comparative roentgenographic study	There was no significant difference in the angle of cervical lordosis and degenerative spinal disease scores between smokers and non-smokers, suggesting no effect of smoking on the cervical spine. (Gore et al., 2006)
Jakoi et al.	2017	Lumbar Intervertebral DDD	Retrospective analysis of a nationwide private insurance database	Smoking had the greatest effect on lumbar spine degeneration compared to any other comorbidities. (Jakoi et al., 2017)
Joswig et al.	2017	Lumbar DDD	Two-center retrospective study	Smoking did not appear to have an effect on patient-reported outcome measures, measuring subjective functional impairment. (Joswig et al., 2017)
Bellitti et al.	2021	DDD	Review article	Smoking is a risk factor for DDD. (Bellitti et al., 2021)
Zhong et al.	2021	Cervical spondylotic myelopathy	Retrospective cohort study	Smoking was not found to be a risk factor for cervical spondylotic myelopathy. (Zhong et al., 2021)
Baucher et al.	2021	Degenerative cervical myelopathy	Review article	The mechanism by which smoking promotes spinal degeneration could be explained by the effects on nicotine on the vascular supply to the IVD, and also by activating the proinflammatory stress response, thus causing damage and leading to DDD. (Baucher et al., 2021)
Abbas et al.	2013	Degenerative LSS	Descriptive study of association between demographic factors, and physical characteristics with degenerative LSS	Smoking was not found to be associated with the diagnosis of degenerative LSS, even though it is a known predictor of the disease. (Abbas et al., 2013)
Ding et al.	2021	TSS	Retrospective study	Smoking was not found to be a risk factor for TSS. (Ding et al., 2021)
Kiraz, M. and Demir, E	2020	Lumbar DDD	Prospective study	Smoking was found to be a significant risk factor for Lumbar DDD, particularly in the L5-S1 spinal levels. (Kiraz and Demir, 2020)
Chen et al.	2018	Cervical DDD	Retrospective study	Smoking was found to exacerbate and accelerate cervical disc degeneration, causing more severe neck and shoulder pain in patients. (Chen et al., 2018)
Lambrechts et al.	2021	Cervical DDD	Retrospective study	Smoking caused increased cervical spinal disc degeneration. (Lambrechts et al., 2021)
Elmasry et al.	2015	IVD degeneration	A finite element study	Smoking tended to affect the AF more than NP in lighter smokers, although for heavy smokers, it caused decreased GAG levels in both the NP and the AF, causing degeneration of the discs. (Elmasry et al., 2015)
Battié et al.	1991	Lumbar Intervertebral DDD	Twin Cohort Study	Smoking was found to have a strong impact on the lumbar discs in this study particularly, as it was compared between twins who were genetically identical. (BATTIÉ et al., 1991)
Doğan et al.	2019	Lumbar DDD	Retrospective study	Cigarette smoking can lead to lumbar intervertebral DDD. (Doğan et al., 2019)
Han et al.	2017	Lumbar Intervertebral DDD	Retrospective study	Smoking did not appear to cause Modic changes in the lumbar discs of patients. (Han et al., 2017)
Huang et al.	2015	LDH	Systematic review	Smoking appeared to promote the occurrence of LDH. (Huang et al., 2015)
Altun, I. and Yuksel, Kz.	2017	IVD degeneration	Experimental study	Maternal smoking before and during pregnancy and before lactation caused increased fibrosis and decreased proteoglycans, leading to increased degeneration in the spine of the new-borns. (Altun and Yuksel, 2017)
Wang et al.	2012	IVD degeneration	Experimental study	Tobacco smoking affects the proteoglycan content in the discs as well the process of replenishing them and collagen. Thus, smoking causes degeneration of the spinal discs. (Wang et al., 2012)
Kwon et al.	2020	Spinal DDD	Retrospective cohort study	Smoking affects the spine in patients and leads to degeneration and increased lower back pain compared to non-smokers. (Kwon et al., 2020)
Saberi et al.	2009	Lumbar spinal disease	Prospective cross-sectional study	Smoking leads to NP dislodgement and subsequent spinal degeneration. (Saberi et al., 2009)
Nakahashi et al.	2019	IVD degeneration	Experimental study	Passive smoking directly affects both the NP and CEP of IVDs, sparing AF. However, the mechanism of action differs in that the architecture and characteristics of NP are damaged by smoking, whereas apoptosis is induced in CEP. (Nakahashi et al., 2019)
Numaguchi et al.	2015	IVD degeneration	Experimental study	Smoking was found to disrupt normal molecular mechanisms by disrupting genes that contributed to the maintenance of the circadian rhythm. As a result, alterations to molecular mechanisms led to the destruction of the IVD. (Numaguchi et al., 2015)
MacDowall et al.	2017	Cervical DDD	Post hoc analysis of a Randomised controlled trial	Smoking did not play a role in non-neurogenic neck pain in people with cervical DDD. (MacDowall et al., 2017)
Khurana, VG	2021	Spondylosis	Literature Review	Smoking not only caused degeneration of the spine and spondylosis, but also postoperative complications and impairment in wound healing. (Khurana, 2021)
Jacobsen et al.	2007	Degenerative lumbar spondylolisthesis	Cross-sectional epidemiological study	Smoking did not play a role in degenerative lumbar spondylolisthesis. (Jacobsen et al., 2007)
Schumann et al.	2010	Lumbar DDD	Multi-center Case-Control Study	The correlation between smoking and LDH was unclear, and according to the study, did not have a clear dose-response relationship. (Schumann et al., 2010)
Yang et al.	2019	LDH	Case-Control Study	Smoking was found to have a greater effect in individuals with certain genes that made them more susceptible to the effects of the chemical components of cigarettes. Certain polymorphisms were also found to be more protective against smoking than others. There was an interesting correlation between smoking and genetic susceptibilities. (Yang et al., 2019)
Snyder et al.	2010	Degenerative spondylolisthesis and spinal stenosis	Cohort study	Spondylolisthesis treatment was not affected by smoking and there were no post-treatment complications. Spinal stenosis surgery can be complicated by smoking including infection and other post-surgical complications. (Snyder et al., 2022)
Sheung-tung, H.	2017	Lumbar disc prolapse, LSS, Cervical myelopathy	Review article	Smoking was responsible for a variety of possible complications following surgery including poor wound healing and greater mortality. Surgery was often indicated in smokers, and they carried a greater risk of developing surgical site infections. (Sheung-tung, 2017)
Sandén et al.	2011	LSS	Cohort study	Poor outcomes and satisfaction post-surgery for LSS was observed in patients who smoked compared to those who did not. (Sandén et al., 2011)
Liu et al.	2021	Cervical laminoplasty for cervical myelopathy	Retrospective Review	Smoking caused a decrease in the range of motion and higher reoperation rates of the cervical spine following cervical laminoplasty. (Liu et al., 2021)
An et al.	1994	Lumbar and Cervical DDD	Retrospective Study	Smoking significantly increased the risk of developing lumbar disc prolapse and cervical disc degeneration in both males and females. (An et al., 1994)
Burkhardt et al.	2020	Cervical fusion and Lumbar DDD	Cohort study	Smoking did not play a role in indicating surgery for lumbar DDD and anterior cervical fusion. (Burkhardt et al., 2020)
Tu et al.	2019	Cervical disc arthroplasty for cervical disc herniation or spondylosis	Retrospective Review	Cervical disc arthroplasty may be a good option for smokers as it had a more improved outcome than non-smokers. (Tu et al., 2019)
Konovalov et al.	2021	Lumbar total disc arthroplasty for DDD	Observational study	Smoking increased post-surgical complication of heterotopic ossification in the spine, but did not affect mortality. (Konovalov et al., 2021)
Smith et al.	2014	Lumbar DDD	Retrospective Review	Smoking affected the recovery of patients post-surgery, with decreased satisfaction and increased pain. (Smith et al., 2014)
Nunley et al.	2013	Cervical DDD	Randomised Controlled Trial	Smoking did not play a role in causing the complication of developing adjacent segment disease following total disc replacement in the cervical spine. (Nunley et al., 2013)
Tetreault et al.	2016	Degenerative cervical myelopathy	Systematic Review	Smoking did not play a role in complications post laminectomy or laminoplasty for degenerative cervical myelopathy. (Tetreault et al., 2016)
Stienen et al.	2016	Lumbar spine surgeries for LDH or LSS	Prospective observational study	Smoking does not impact the response of a patient to surgery but does delay healing, potentially causing the need to undergo surgery again. (Stienen et al., 2016)
Wang et al.	2021	Cervical DDD	Retrospective single-center cohort study	Smoking causes poorer outcomes following hybrid surgery for multilevel cervical disc disease, including poor fusion and increased bone loss. (Wang et al., 2021)
Behrend et al.	2012	IVD disease	Prospective study	There is a strong association between smoking and pain in people undergoing surgery, which can be improved with smoking cessation. (Behrend et al., 2012)
Asher et al.	2017	Lumbar DSD	Retrospective analysis of prospectively collected data	Smokers were more likely to undergo surgery or decompression for their spinal disease and reported greater pain at baseline and following surgery than non-smokers. (Asher et al., 2017)
Bydon et al.	2015	Laminectomy for Lumbar spondylosis	Retrospective Review	Smoking was found to be a strong predictor of reoperation after surgeries for lumbar spondylosis. (Bydon et al., 2015)
Nakhla et al.	2018	Spondylolisthesis	Retrospective Review	Smoking appears to cause wound complications only following certain surgeries and not others, suggesting that certain surgeries may be a better option for smokers. Smoking however, still was a predictor of infection regardless of which fusion option was chosen. (Nakhla et al., 2018)
Patel et al.	2020	Lumbar Spondylolisthesis	Prospective Study	There appeared to not be a great difference in response to the surgery for grade 1 lumbar spondylolisthesis between smokers and non-smokers, although smoking appears to decrease the chances of achieving minimum clinically important difference in ODI since smokers have a low baseline ODI to begin with. (Patel et al., 2020)
Goyal et al.	2020	Lumbar decompression for spinal stenosis/disc herniations	Retrospective cohort study	Smoking status was not a predictor of outcome following lumbar decompression. (Goyal et al., 2020)
Ngo et al.	2017	IVD degeneration	Experimental study	Disc degeneration due to smoking is increased by activating ADAMTS5, which causes pathological release of aggrecan and also induced inflammatory pathways, causing further disc damage. (Ngo et al., 2017)
Jing et al.	2020	IVD degeneration	Experimental study	Smoking appears to cause the activation of apoptosis of AF through the mitochondrial pathway, induced by cadmium accumulation in the body. (Jing et al., 2020)
Cong et al.	2010	IVD degeneration	Experimental study	Smoking tended to impact certain alleles more than the other and to different extents, suggesting yet again a relationship between susceptible genes and smoking on DDD. (Cong et al., 2010)
Lo et al.	2021	IVD degeneration	Experimental study	Nicotine causes the degeneration of the IVDs by impacting the IGF-1 pathway, which causes chondrocyte reduction as well as a decrease in chondrogenic indicator levels. (Lo et al., 2021)
Luo et al.	2020	LDH	Case-Control Study	Another study showing the impact of smoking on people who already have genes susceptible to developing LDH, showcasing a clear increase in the trend. (Luo et al., 2020)
Gulati et al.	2015	LSS	Multi-center Observational registry-based study	Smokers with LSS had decreased improvement at 1 year following microcompression, greater pain and decreased number of smokers were able to reach the minimal clinically important difference for spinal degeneration. (Gulati et al., 2015)
Hadley, M and Reddy, S	1997	DSD	Review article	Smokers tend to cause both preoperative and post-operative issues, including poorer outcomes and bony degradation. (Hadley and Reddy, 1997)
Connor et al.	2020	DSD	Retrospective Database Study	Smoking causes a greater risk of readmission 90 days post-surgery, which might be a factor to consider prior to electing for surgery in these patients. (Connor et al., 2020)
Grisdela et al.	2017	Cervical DDD	Retrospective analysis	Tobacco use increased the chances of undergoing surgery, in both patients with or without myelopathy and disc disease. Hence, smoking is an independent predictor of surgery. (Grisdela et al., 2017)
Nasto et al.	2014	IVD degeneration	Experimental study	Spinal disc degeneration was highly impacted by smoking, where the main factors responsible for the maintenance of the discs were destroyed. (Nasto et al., 2014)
Holm, S and Nachemson A	1988	IVD degeneration	Experimental study	Smoking caused impairment of nutritional supply and oxygen supply to the discs, leading to impaired aerobic respiration, a consequent build-up of lactate, and degeneration of the disc. (Holm and Nachemson, 1988)
Fogelholm, R. R and Alho, A. V	2001	IVD degeneration	Review article/Medical hypotheses	Smoking contributes to the degeneration of the spinal disc, which is responsible for causing debilitating lower back pain. The authors further hypothesise that “high serum proteolytic activity of cigarette smokers gets access to a previously degenerated neovascularized disc and speeds up the degenerative process”. (Fogelholm and Alho, 2001)

We also highlight the fact that smoking can contribute to ‘non-union’ (Pearson et al., 2016) and ‘delayed union’ of the spine following spinal fusion leading to failure of the procedure. This prompts the fact that patients should be strongly advised to quit smoking before they opt to undergo spinal fusion as elucidated by the recent meta-analysis by Yang Li et al. (2021)

### 4.3 Limitations of the studies

Cigarette smoke consists of a multitude of toxic substances, which can cause extensive damage in any of the body’s systems, as a result of which it is considered a risk factor for most diseases. Several studies discussed above in results consider smoking to be a risk factor in the development of DSD. Considering all the studies discussed however, it can potentially be suggested that smoking only primarily affects the lumbar spine compared to the cervical spine or thoracic spine. However, this cannot be concluded for certain as there are only a smaller number of studies where smoking was found not to be a significant risk factor for cervical DSD (MacDowall et al., 2017; Zhong et al., 2021; Gore et al., 2006; Nunley et al., 2013; Tetreault et al., 2016). Thus, more studies looking into the effects of smoking on the cervical spine are thus required to define a definite relationship between them.

In the study by Gulati et al., the limitation lies in the fact that the relationship between smoking and post-operative complications in lumbar DDD patients was unable to be confirmed as causal, as potential confounding factors may play a role, which they had been unable to establish (Gulati et al., 2015). Furthermore, additional variables such as age and gender may have had some role in explaining the outcome of the patient and were not controlled for completely (Gulati et al., 2015). They also faced some missing information, which rendered them unable to create a complete dose-response curve (Gulati et al., 2015). Nasto et al. utilised mice to showcase the genetic changes that can potentially be caused by cigarette smoke, leading to bone damage and degeneration (Nasto et al., 2014). The mice are, however, exposed at a relatively early stage of development, which could lead to greater damage than being exposed as adults, hence limiting generalisation of results to adults (Nasto et al., 2014).

The major drawback to most studies was the absence of repeat studies and details in terms of elucidating the mechanism. However, it appears that these various studies may together stand as sufficient to showcase the overall effects on the IVDs, even though the exact mechanism of action hypothesised may differ amongst the studies. We have focussed on the evidence for tobacco smoking and have excluded other substances abused that can be inhaled in the form of smoking.

## 5 Conclusion

Tobacco cigarette smoking is a known risk factor for a plethora of debilitating diseases. Over the last few decades, there has been increasing amounts of research now finding an association with smoking and DSD. In this review, we attempted to consolidate the multitude of research looking at the effect of smoking on spinal degeneration. It can be deduced that smoking appears to be a significant risk factor for lumbar DSDs, although some studies also suggest its role in causing cervical spine degeneration. There is unfortunately insufficient research on the effect of smoking on the thoracic spine based on the literature search that was conducted, as a result of which it is difficult to identify the role of smoking for certain in the degeneration of the thoracic spine. Additionally, smoking appears to lead to worse outcomes and potential complications post-surgery and in several cases, can be the reason for surgery. It also contributes to increased pain perception and poorer subjective response from patients following surgery. Further research into the cost-benefit analysis of funding smoking cessation programs pre- and post-operatively to address these difficulties for patients would also be very beneficial to prevent further complications and also to economically benefit the healthcare industry by saving cost of treatment. Several researchers have attempted to identify the exact mechanism by which smoking leads to spinal damage. However, given the number of harmful ingredients in cigarettes and the various effects each of them have on the body, several mechanisms were identified, which could explain the course of degeneration in the spine and the IVDs. Ultimately, smoking appears to have a causal relationship with the onset of spinal degeneration, as well as complications post-treatment and surgery for the same. Further studies, both retrospective and prospective, are needed to identify the effect of smoking on the cervical and thoracic spine pathology, as well as to identify if smoking cessation can reverse the damage done to the spine.

## Funding

None.

## Declaration of competing interest

The authors declare no competing interests.

The authors have no relevant financial or non-financial interests to disclose.

All authors contributed to the production of this manuscript equally. All authors have reviewed the manuscript and are happy with its contents.
